# First-Trimester Serum Acylcarnitine Levels to Predict Preeclampsia: A Metabolomics Approach

**DOI:** 10.1155/2015/857108

**Published:** 2015-06-04

**Authors:** Maria P. H. Koster, Rob J. Vreeken, Amy C. Harms, Adrie D. Dane, Sylwia Kuc, Peter C. J. I. Schielen, Thomas Hankemeier, Ruud Berger, Gerard H. A. Visser, Jeroen L. A. Pennings

**Affiliations:** ^1^Department of Obstetrics, Wilhelmina Children's Hospital, University Medical Centre Utrecht (UMCU), 3508 AB Utrecht, Netherlands; ^2^Leiden Academic Centre for Drug Research, Division of Analytical Biosciences, Leiden University, 2300 RA Leiden, Netherlands; ^3^Discovery & Exploratory BA, Pharmacokinetics, Dynamics & Metabolism, Discovery Sciences, Janssen Pharmaceutica, Beerse, Belgium; ^4^Centre for Infectious Diseases Research, Diagnostics and Screening (IDS), National Institute for Public Health and the Environment (RIVM), 3720 BA Bilthoven, Netherlands; ^5^Netherlands Metabolomics Centre, 3501 DE Utrecht, Netherlands; ^6^Centre for Health Protection (GZB), National Institute for Public Health and the Environment (RIVM), 3720 BA Bilthoven, Netherlands

## Abstract

*Objective*. To expand the search for preeclampsia (PE) metabolomics biomarkers through the analysis of acylcarnitines in first-trimester maternal serum. *Methods*. This was a nested case-control study using serum from pregnant women, drawn between 8 and 14 weeks of gestational age. Metabolites were measured using an UPLC-MS/MS based method. Concentrations were compared between controls (*n* = 500) and early-onset- (EO-) PE (*n* = 68) or late-onset- (LO-) PE (*n* = 99) women. Metabolites with a false discovery rate <10% for both EO-PE and LO-PE were selected and added to prediction models based on maternal characteristics (MC), mean arterial pressure (MAP), and previously established biomarkers (PAPPA, PLGF, and taurine). *Results*. Twelve metabolites were significantly different between EO-PE women and controls, with effect levels between −18% and 29%. For LO-PE, 11 metabolites were significantly different with effect sizes between −8% and 24%. Nine metabolites were significantly different for both comparisons. The best prediction model for EO-PE consisted of MC, MAP, PAPPA, PLGF, taurine, and stearoylcarnitine (AUC = 0.784). The best prediction model for LO-PE consisted of MC, MAP, PAPPA, PLGF, and stearoylcarnitine (AUC = 0.700). *Conclusion*. This study identified stearoylcarnitine as a novel metabolomics biomarker for EO-PE and LO-PE. Nevertheless, metabolomics-based assays for predicting PE are not yet suitable for clinical implementation.

## 1. Introduction

Preeclampsia (PE) is a hypertensive complication that occurs in approximately 3% of all pregnancies and may lead to poor pregnancy outcomes of both mother and fetus [1, 2]. It is thought that in women with PE a complex interaction between placental factors, maternal constitutional factors, and pregnancy-specific vascular and immunological adaptation occurs already in the first trimester of their pregnancy [3–5]. The clinical manifestations of PE, such as high blood pressure and proteinuria, are only terminal features of this cascade of events. Therefore, early recognition of women at risk and timely intervention ahead of clinical onset might enable tailored pregnancy care and better pregnancy outcomes.

In the last decade, several studies have been performed focusing on the detection of markers to predict preeclampsia [6]. At present the most promising marker for the prediction of PE is an ultrasound Doppler measurement of the uterine arteries [6, 7]. However, this approach has technical, logistical, and financial barriers for implementation in a population screening setting. This stretches the need for additional (bio)markers and a targeted approach that can be used for this purpose is metabolomics analysis of maternal blood.

In a previous publication by our group we provided an overview of all first-trimester studies that used metabolomics techniques in maternal blood for the early prediction of PE [8]. In that study, we also performed a metabolomics experiment in a large cohort of women with and without PE, which indicated three potential biomarkers for the early prediction of PE. Since then, only one other study has been published on this subject with comparable results; metabolomic profiles of ten women with PE were different from those of ten normal pregnant women [9]. However, up until today the search for metabolomic biomarkers has not yet revealed the perfect combination of metabolites to accurately predict PE in a clinical setting.

Several pathophysiological pathways probably contribute to the phenotype of PE. For example, PE is associated with abnormal lipid metabolism, including fatty acid oxidation metabolism [10]. Fatty acids play an important role during pregnancy as metabolic fuel for the placenta [11]. When fatty acid oxidation is defective or diminished an increase in plasma acylcarnitine levels can be observed. It has been shown that maternal acylcarnitine levels are indeed increased in third-trimester PE pregnancies compared to healthy controls [12]. The aim of the current study was to expand the search for new biomarkers using metabolomic techniques by the analysis of acylcarnitines in first-trimester maternal serum from women with and without PE.

## 2. Methods

### 2.1. Study Population and Outcome Measures

This was a nested case-control study derived from a large cohort of women participating in the routine Dutch first-trimester prenatal screening between 2007 and 2009, including singleton pregnancies with a delivery >24 weeks of gestational age (GA). The study design and population have extensively been described elsewhere [8, 13]. In short, baseline characteristics of the study population, such as maternal age, sample date, GA at sampling, maternal weight, and smoking status, were recorded by a midwife or gynecologist. Maternal blood was drawn at 8^+0^–13^+6^ weeks of GA and stored at −80°C until metabolomics analysis.

Pregnancy outcomes, including chromosomal abnormalities, date of birth, birth weight, and the presence of hypertensive pregnancy complications (PE, HELLP syndrome, or pregnancy induced hypertension), were collected through self-reporting of the participating women. The self-reported diagnoses of PE were confirmed through hospital records and data on maternal characteristics, that is, medical history, parity, weight, height, and blood pressure, were subsequently collected. The control group, consisting of 500 women who delivered phenotypically and chromosomally normal neonates at term (37^+0^–42^+0^ weeks) without developing any pregnancy complication, was randomly selected without matching. For these pregnancies, outcomes were also confirmed and missing maternal characteristics and blood pressure values were collected.

PE was defined as gestational hypertension beyond 20 weeks of GA in previously normotensive women with a systolic blood pressure ≥140 mm Hg and/or diastolic blood pressure ≥90 mm Hg and the presence of proteinuria of ≥300 mg in 24-hour collection, according to the criteria of the International Society for the Study of Hypertension in Pregnancy [14]. Early-onset-PE (EO-PE) was defined as PE in pregnancies where delivery took place before 34 weeks of GA; all other PE cases were considered late-onset-PE (LO-PE). Mean arterial pressure (MAP) was calculated from the formula: diastolic blood pressure + 1/3 *∗* (systolic blood pressure − diastolic blood pressure).

### 2.2. Sample Analysis

As part of a previous study on the prediction of PE [13], pregnancy associated plasma protein A (PAPPA) and placental growth factor (PlGF) were measured in maternal serum using AutoDELFIA time resolved assays (Perkin Elmer, Turku, Finland).

The acylcarnitine platform is an UPLC-MS/MS based method that allows for the separation and quantification of several important isomers of acylcarnitine species as well as trimethylamine-N-oxide, choline, betaine, deoxycarnitine, and carnitine. Ten microliters (*μ*L) of each serum sample was spiked with an internal standard solution containing deuterated analogs of eight target compounds spreading the entire polarity range, followed by deproteination by addition of MeOH. The supernatant was transferred to an autosampler vial. The vials were transferred to an autosampler tray and cooled to 10°C until injection. One *μ*L of the reaction mixture was injected into the UPLC-MS/MS system.

An ACQUITY UPLC system with autosampler (Waters, Etten-Leur, Netherlands) was coupled online with a Xevo Tandem Quadrupole (TQ) mass spectrometer (Waters) operated using Masslynx data acquisition software (version 4.1; Waters). The samples were analyzed by UPLC-MS/MS using an Accq-Tag Ultra column (Waters). The Xevo TQ was used in the positive-ion electrospray mode and all analytes were monitored in Multiple Reaction Monitoring (MRM) using nominal mass resolution.

Acquired data were evaluated using TargetLynx software (Waters), by integration of assigned MRM peaks and normalization using proper internal standards. The closest-eluting internal standard was employed. Blank samples were used to correct for background, and in-house developed algorithms were applied using the pooled QC samples to compensate for shifts in the sensitivity of the mass spectrometer over the batch.

### 2.3. Statistical Analysis

We used a similar statistical procedure as in our previous study [8]. For this approach, the data set was divided into sets for training, testing (evaluation), and validation, respectively. For each group (i.e., controls, EO-PE, and LO-PE), samples were randomly assigned to the training (40%), test (30%), or validation (30%) set. Overall sample assignment was as follows: training set: 200 controls, 27 EO-PE women, and 40 LO-PE women; test set: 150 controls, 20 EO-PE women, and 30 LO-PE women; validation set: 150 controls, 21 EO-PE women, and 29 LO-PE women. Sample assignment was made before the data analysis of our previous study and therefore the three subsets basically correspond to the previous study, except for four samples that failed during analysis (one control and one EO-PE woman in the training set, one control and one LO-PE woman in the test set).

After this random assignment we confirmed that there were no significant differences in maternal characteristics (i.e., medical records, parity, weight, and length) between the three sets. Maternal characteristics were used to calculate prior risks of EO-PE and LO-PE as described earlier [8]. Next, metabolite data were compared between controls and either EO-PE or LO-PE women, using Student's *t*-test on log-transformed data. Values were corrected for multiple testing by calculating the false discovery rate (FDR). Metabolites with a FDR <10% for both EO-PE and LO-PE women were selected for fitting PE prediction models, using logistic regression. Training models were calculated on training set data (controls and either EO-PE or LO-PE cases) for prior risks, log-MAP, and log-transformed data for significant metabolites as well as several metabolite combinations. Models were then tested on the test set data for the corresponding metabolites. Models were evaluated based on their predicted detection rate (DR; sensitivity) in the test set for a fixed 10% false positive rate (FPR; 1 − specificity) as well as on the area under the curve (AUC). Markers were considered useful biomarkers if adding them to the baseline model (prior risk + MAP) helped improve the DR as well as AUC.

The model with the best performance was validated on the validation set. Finally, this model was compared and combined with data of log-transformed levels of PAPPA and PlGF as well as log-transformed levels of taurine, three predictive markers for PE for which data was obtained in previous studies by our group [8, 13]. As before, risk prediction models were calculated on training set data, but since these markers have already been established they were only applied for the validation set.

Statistical analyses were performed using SPSS (release 20.0; Chicago, IL), SAS software package (release 9.2; SAS Institute, Cary, NC, USA), and R programming language version 3.0 (http://www.r-project.org/).

### 2.4. Ethics Statement

This study has been approved by the Scientific Ethical Committee of the University Medical Centre Utrecht, Netherlands (protocol number: 11-002). All participants in this study have provided written informed consent.

## 3. Results

### 3.1. Pregnancy Characteristics

The baseline characteristics of our study population are shown in Table 1. Women with EO-PE and LO-PE had higher BMI (24.7 kg/m^2^, *p* < 0.001 and 23.7 kg/m^2^, *p* = 0.005, resp.) and were more often nulliparous (80.9%, *p* < 0.001 and 72.7%, *p* < 0.001, resp.) compared to controls. Also, multiparous women with PE more often had a history of hypertensive pregnancy disorders (EO-PE 30.8%, *p* = 0.009 and LO-PE 37.0%, *p* < 0.001) compared to multiparous controls. Perinatal outcomes differed between groups; both in the EO-PE and in LO-PE group more women delivered prematurely and birth weight centiles were lower.

### 3.2. Metabolite Preselection

We analyzed 24 metabolites for statistically significantly different levels between controls and cases of both EO-PE and LO-PE. For EO-PE, 12 metabolites were significantly different between EO-PE women and controls, with effect levels ranging from an 18% decrease to a 29% increase. For LO-PE, 11 metabolites were significantly different; effect sizes for these metabolites ranged from an 8% decrease to a 24% increase. Although effect sizes for the LO-PE group were typically smaller, the number of significant metabolites was comparable and nine metabolites were significant for both comparisons (Table 2).

Concentrations of some of the selected metabolites were significantly correlated; hexanoylcarnitine, octanoylcarnitine, decenoylcarnitine, and decanoylcarnitine were highly correlated (*R* > 0.8; Table 3).

### 3.3. Model Selection

Prediction models were fitted based on the training set, using the prior risk, MAP, and one or more of the significant metabolites. A model with only prior risk and MAP was used as a baseline model; subsequently which metabolites improved the prediction accuracy was studied.

Comparison of the models, regarding their performance on the test set, indicated that for EO-PE only one model improved the DR, that is, the model containing prior risk, MAP, and stearoylcarnitine. This model showed a DR of 88% (95% CI 63–100%) at a FPR of 10%, which is a 7% gain on the baseline model (Table 4). For LO-PE, three models improved the DR, namely, those based on prior risk and MAP combined with octenoylcarnitine, linoleylcarnitine, and stearoylcarnitine, respectively (Table 4). Of these, the model with stearoylcarnitine showed the highest DR (54%; 95% CI 29–79%) compared to the baseline model (DR 46%; 95% CI 29–67%). The model with octenoylcarnitine did not increase the AUC, so this marker was not used in further modeling. For the two remaining metabolites, the performance of their combined use was also determined, which led to a DR of 50% (95% CI 25–75%) and an AUC of 0.815 (Table 4). Since this did not improve the model with prior risk + MAP + stearoylcarnitine (AUC 0.820), the latter model was selected for validation.

### 3.4. Validation

**Figure 1:**
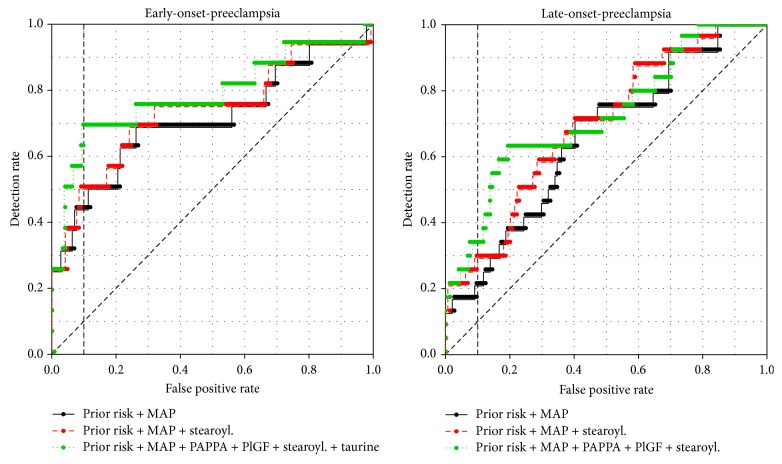
Receiver operating characteristic (ROC) curves for validated models. Black line: prior risk of early-onset- or late-onset-preeclampsia based on maternal characteristics and mean arterial pressure (MAP). Red line: prior risk of early-onset- or late-onset-preeclampsia combined with MAP and stearoylcarnitine. Green line: prior risk of early-onset- or late-onset-preeclampsia combined with MAP, stearoylcarnitine, and other serum markers.

When the selected models were applied on the validation set, the EO-PE model showed a DR of 50% (95% CI 25–70%), which was an improvement upon the DR of 45% (95% CI 25–70%) obtained for the EO-PE baseline model on the validation set (Table 4, Figure 1). The LO-PE model resulted in a DR of 29% (95% CI 8–46%), which was also an improvement upon the DR of 21% (95% CI 4–42%) for the LO-PE baseline model (Table 4, Figure 1). These findings validate the prediction models and, moreover, confirm the selection of stearoylcarnitine as a biomarker for both EO- and LO-PE.

To assess the added value of stearoylcarnitine as part of serum biochemical analyses for PE risk prediction, the obtained models were compared to models based on established serum markers PAPPA and PlGF. This comparison included assessing these models with and without stearoylcarnitine. For LO-PE, the model using prior risk, MAP, PAPPA, and PlGF gave a DR of 27%, which is higher than the model with only prior risk and MAP but lower than the model using prior risk, MAP, and stearoylcarnitine. Adding stearoylcarnitine to the established marker model improved the DR to 32% (AUC 0.700) (Table 4; Figure 1) as the best overall model for LO-PE.

For EO-PE, the model based on prior risk, MAP, PAPPA, and PlGF gave a DR of 56%, which is higher than our stearoylcarnitine-based model. Adding stearoylcarnitine to this model did not improve the DR but increased the AUC from 0.727 to 0.751. Adding taurine to this model, as an additional, previously identified metabolomics marker for EO-PE, improved the DR to 69% (AUC 0.784) (Table 4; Figure 1) as the best overall model for EO-PE.

## 4. Discussion 

In this study, we used UPLC-MS/MS based metabolomics to determine the predictive value of acylcarnitines in first-trimester serum from women who later developed EO-PE or LO-PE. Despite research progress in the last decade, the etiology of PE is not yet completely understood. For example, it is not clear to what extent EO-PE can be attributed to the same pathophysiological processes as LO-PE, whether these are distinct disease entities, or whether they merely represent different points on a continuous scale. This is reflected in literature data on PE biomarkers. Some biomarkers are predictive for only EO-PE or LO-PE, whereas others are predictive for both. From a clinical perspective, however, a difference is usually made between the two due to the associated disease severity. In our search for PE biomarkers, metabolites suitable for prediction of both EO- and LO-PE would be preferable from a general screening and counseling perspective, possible treatment decisions, and laboratory workflow. In this study, we therefore kept the distinction between EO- and LO-PE, as in previous studies on this cohort [8, 13], but with an aim of identifying markers predictive for both types of PE.

PE is a multifactorial and heterogeneous disease, with factors involved being impaired placentation, vascular remodeling, and immunological adaptation. Therefore, metabolomics might offer advantages compared to other “omics” methods such as proteomics and genomics, since it is more targeted to the final downstream products of gene and protein expression changes, which allows for establishing a phenotypic signature of causes, manifestations, and pathways of disease [15].

In this study, we found considerable overlap between the metabolomics markers with significantly different serum concentrations between controls and EO-PE or LO-PE women, respectively. This indicates that acylcarnitine levels are related to a common etiological factor for EO- and LO-PE. For this reason, as well as the reasons described above, nine markers predictive for both types of PE were selected for further prediction modeling. Among these nine markers, various degrees of correlation were found. Interestingly, correlations were higher between acylcarnitines with similar carbon chain length, especially hexa-, octa-, and decaones. The most promising marker (stearoylcarnitine) was not highly correlated with any of the other metabolites. This can probably explain why this marker on itself was found to add significant value for both types of PE in prediction modeling, using a training and test set. The potential value of stearoylcarnitine was subsequently confirmed in the validation set. As the modeling for early- and late-onset-PE was executed separately, it is interesting to find that a single marker is predictive for both types of PE, that is, the entire clinical range, which would fit in with our aims.

Unfortunately, the detection rates obtained with stearoylcarnitine in the validation set are yet too low to be clinically applicable. Including this marker in a panel with previously identified markers showed that stearoylcarnitine added some predictive value to the protein model (prior risk, MAP, PAPPA, and PlGF). However, for EO-PE this improvement was small compared to the additional predictive value of the metabolomics marker taurine, which we identified earlier as a biomarker for EO-PE only [8].

Acylcarnitines have been previously investigated as potential biomarkers for PE in the first [16], second [17], and third [12] trimester of pregnancy, respectively. Notably, these studies as well as ours report different metabolomics biomarkers for PE. Partially, these will be attributable to the sample material used, that is, serum or plasma, as well as differences due to gestational age and accordingly disease stage. Additionally, variations in study size and metabolomics protocol and analysis can contribute to these differences. In this respect, a particular strength of our study is its large sample size as well as its use of first-trimester serum, that is, ahead of PE diagnosis. Moreover, our study was large enough to allow internal validation of prediction models on a separate subset of the data. However, these prediction models should be externally validated before being suitable for clinical implementation. Preferably, a biomarker for PE should be predictive throughout the entire first trimester. However, due to several processes in early pregnancy, biomarkers might be predictive at different gestational weeks. We did not perform a subanalysis among gestational weeks; however, most of our samples were taken in week 11 or 12, thus narrowing the first-trimester gestational age range. Also, there was a small difference in gestational age at sample between preeclampsia cases and uncomplicated controls (3 days; Table 1). Although this difference was statistically significant in the entire study group, we did not observe any differences within our test, training, and validation set and therefore do not expect this to have affected our results.

## 5. Conclusions

In conclusion, acylcarnitines in general do not seem to add significantly to the prediction of PE, probably indicating that, overall, these metabolites are not involved in its underlying pathophysiological pathways. Our study did identify stearoylcarnitine as a potential novel metabolomics biomarker for both EO-PE and LO-PE. Nevertheless, metabolomics-based assays for predicting PE are not yet suitable for clinical implementation.

## Figures and Tables

**Table 1:** Baseline characteristics of the study population (adapted from Kuc et al. [13]).

Characteristics	Controls	EO-PE	LO-PE
	*n* = 500	*n* = 68	*n* = 99
Maternal age (y)	33 (30–35)	34 (30–37)	33 (30–36)
Maternal weight (kg)	65.5 (60.0–73.0)	70.0 (62.0–81.5)^*∗*^	67.5 (62.0–75.0)
Maternal BMI (kg/m^2^)	22.8 (20.7–24.8)	24.7 (21.9–29.3)^*∗*^	23.7 (21.3–26.5)^*∗*^
Nulliparity	233 (46.6)	55 (80.9)^*∗*^	72 (72.7)^*∗*^
Smoking	21 (4.2)	8 (11.8)^*∗*^	6 (6.1)
Assisted reproduction	0 (0)	3 (4.4)^*∗*^	8 (8.1)
Gestation at sampling (days)	88 (84–91)	85 (76–89)^*∗*^	85 (79–89)^*∗*^
History of hypertensive pregnancy disorders^†^	4 (1.5)	4 (30.8)^*∗*^	10 (37.0)^*∗*^
Gestational age at birth (wk)	40 (39–41)	31 (30–32)^*∗*^	37 (36–39)^*∗*‡^
Birth weight (grams)	3544 (3243–3800)	1300 (1045–1609)^*∗*^	2650 (2130–3110)^*∗*‡^
Birth weight centile	57.0 (33.1–78.4)	25.0 (13.4–50.4)^*∗*^	13.8 (3.8–46.0)^*∗*‡^
Fetal gender, male	244 (48.8)	34 (49.7)	53 (53.5)

Values are presented as median and interquartile ranges or numbers and percentages. Mann-Whitney *U* test and chi-square test, both with post hoc Bonferroni correction (adjusted significance value *p* < 0.007), were used for statistical comparison between EO-PE women, LO-PE women, and controls. ^†^Percentage based on multiparous women only. ^*∗*^Significantly different from controls. ^‡^Significantly different between EO-PE and LO-PE. EO-PE: early-onset-preeclampsia; LO-PE: late-onset-preeclampsia; BMI: body mass index.

**Table 2:** List of acylcarnitines that were measured in this study with accompanying fold changes, *p* values, and false discovery rates (FDR).

Metabolite	EO-PE versus controls			LO-PE versus controls			Selection
	Ratio	*p* value	FDR	Ratio	*p* value	FDR	
Choline	0.983	0.824	0.827	0.998	0.971	0.971	
Betaine	1.013	0.788	0.827	1.032	0.406	0.513	
Deoxycarnitine	0.944	0.082	0.140	0.971	0.265	0.374	
Carnitine	1.009	0.758	0.827	1.045	0.094	0.173	
Acetylcarnitine	0.947	0.501	0.707	0.910	0.136	0.218	
Propionylcarnitine	0.910	0.055	0.102	0.996	0.928	0.971	
Isobutyrylcarnitine	0.822	0.006	0.015	0.958	0.436	0.523	EO-PE
2-Methylbutyroylcarnitine	1.016	0.761	0.827	1.041	0.299	0.398	
Isovalerylcarnitine	1.038	0.613	0.774	1.110	0.083	0.166	
Hexanoylcarnitine	1.234	0.008	0.016	1.114	0.044	0.095	Both
Octenoylcarnitine	1.281	<0.001	0.004	1.240	<0.001	0.004	Both
Octanoylcarnitine	1.378	0.001	0.005	1.201	0.020	0.054	Both
Nonaylcarnitine	0.903	0.242	0.363	0.967	0.644	0.736	
Decenoylcarnitine	1.253	0.001	0.005	1.198	0.001	0.004	Both
Decanoylcarnitine	1.423	0.001	0.004	1.200	0.031	0.074	Both
Dodecenoylcarnitine	1.272	0.003	0.008	1.162	0.015	0.050	Both
Lauroylcarnitine	1.328	0.002	0.007	1.108	0.152	0.228	EO-PE
Tetradecenoylcarnitine	1.493	<0.001	0.001	1.248	0.017	0.052	Both
Myristoylcarnitine	1.032	0.558	0.744	0.921	0.121	0.208	
Hexadecenoylcarnitine	1.189	0.007	0.016	0.996	0.954	0.971	EO-PE
Linoleylcarnitine	0.849	0.004	0.011	0.851	0.002	0.007	Both
Palmitoylcarnitine	0.992	0.827	0.827	0.896	0.002	0.007	LO-PE
Oleylcarnitine	0.922	0.089	0.143	0.856	0.001	0.007	LO-PE
Stearoylcarnitine	0.829	<0.001	0.001	0.842	<0.001	<0.001	Both

Fold changes (ratio) were calculated based on log-transformed data and Student's *t*-tests were used for comparison. *p* values were corrected for multiple testing by calculating FDR. Metabolites with a FDR <10% for both EO-PE and LO-PE were selected for fitting prediction models. EO-PE: early-onset-preeclampsia; LO-PE: late-onset-preeclampsia.

**Table 3:** Correlation between markers selected for prediction modeling. Please note that the table is symmetrical and each correlation pair is presented twice.

	Hexanoyl- carnitine	Octenoyl- carnitine	Octanoyl- carnitine	Decenoyl- carnitine	Decanoyl- carnitine	Dodecenoyl- carnitine	Tetradecenoyl- carnitine	Linoleyl- carnitine	Stearoyl- carnitine
Hexanoylcarnitine	1	0.26	0.94	0.82	0.94	0.73	0.58	0.16	0.19
Octenoylcarnitine	0.26	1	0.18	0.50	0.19	0.35	0.27	0.28	0.10
Octanoylcarnitine	0.94	0.18	1	0.81	0.99	0.69	0.57	0.12	0.14
Decenoylcarnitine	0.82	0.50	0.81	1	0.83	0.82	0.60	0.28	0.16
Decanoylcarnitine	0.94	0.19	0.99	0.83	1	0.75	0.61	0.11	0.15
Dodecenoylcarnitine	0.73	0.35	0.69	0.82	0.75	1	0.72	0.22	0.21
Tetradecenoylcarnitine	0.58	0.27	0.57	0.60	0.61	0.72	1	0.20	0.18
Linoleylcarnitine	0.16	0.28	0.12	0.28	0.11	0.22	0.20	1	0.59
Stearoylcarnitine	0.19	0.10	0.14	0.16	0.15	0.21	0.18	0.59	1

**Table 4:** Detection rates (DR) at a fixed 10% false positive rate (FPR) and *c*-statistics (AUC) for various prediction models based on selected markers. Only models that improved the performance of the baseline model (prior risk + MAP) were used in the validation set.

Model	Training set		Test set		Validation set	
EO-PE	DR (95% CI)	AUC	DR (95% CI)	AUC	DR (95% CI)	AUC

Prior risk + MAP	55 (35–80)	0.879	81 (50–100)	0.914	45 (25–70)	0.720
Prior risk + MAP + hexanoylcarnitine	60 (35–80)	0.887	69 (38–94)	0.897	—	—
Prior risk + MAP + octenoylcarnitine	60 (35–80)	0.875	75 (50–94)	0.913	—	—
Prior risk + MAP + octanoylcarnitine	60 (35–80)	0.881	75 (44–100)	0.909	—	—
Prior risk + MAP + decenoylcarnitine	60 (35–80)	0.879	75 (44–100)	0.915	—	—
Prior risk + MAP + decanoylcarnitine	60 (35–80)	0.882	75 (44–100)	0.908	—	—
Prior risk + MAP + dodecenoylcarnitine	55 (35–80)	0.883	75 (44–100)	0.910	—	—
Prior risk + MAP + tetradecenoylcarnitine	60 (40–80)	0.885	75 (44–94)	0.905	—	—
Prior risk + MAP + linoleylcarnitine	55 (35–80)	0.879	81 (50–100)	0.915	—	—
Prior risk + MAP + stearoylcarnitine	60 (35–80)	0.877	88 (63–100)	0.935	50 (25–70)	0.747
Prior risk + MAP + PAPPA + PlGF	58 (32–84)	0.884	—	—	56 (31–81)	0.727
Prior risk + MAP + PAPPA + PlGF + stearoylcarnitine	68 (42–84)	0.883	—	—	56 (31–81)	0.751
Prior risk + MAP + PAPPA + PlGF + stearoylcarnitine + taurine	68 (42–89)	0.878	—	—	69 (38–88)	0.784

LO-PE	DR (95% CI)	AUC	DR (95% CI)	AUC	DR (95% CI)	AUC

Prior risk + MAP	46 (23–71)	0.816	46 (29–67)	0.790	21 (4–42)	0.652
Prior risk + MAP + hexanoylcarnitine	49 (29–69)	0.824	46 (21–71)	0.787	—	—
Prior risk + MAP + octenoylcarnitine	54 (31–71)	0.822	50 (25–75)	0.788	—	—
Prior risk + MAP + octanoylcarnitine	49 (29–69)	0.820	42 (21–71)	0.784	—	—
Prior risk + MAP + decenoylcarnitine	49 (31–69)	0.823	42 (25–67)	0.785	—	—
Prior risk + MAP + decanoylcarnitine	46 (29–69)	0.818	46 (21–67)	0.785	—	—
Prior risk + MAP + dodecenoylcarnitine	46 (26–66)	0.815	42 (25–67)	0.790	—	—
Prior risk + MAP + tetradecenoylcarnitine	43 (26–66)	0.821	42 (21–63)	0.789	—	—
Prior risk + MAP + linoleylcarnitine	46 (23–71)	0.816	50 (29–71)	0.799	—	—
Prior risk + MAP + stearoylcarnitine	49 (26–77)	0.828	54 (29–79)	0.820	29 (8–46)	0.692
Prior risk + MAP + stearoylcarnitine + linoleylcarnitine	57 (34–77)	0.829	50 (25–75)	0.815	—	—
Prior risk + MAP + PAPPA + PlGF	55 (36–70)	0.825	—	—	27 (9–55)	0.688
Prior risk + MAP + PAPPA + PlGF + stearoylcarnitine	58 (36–79)	0.833	—	—	32 (9–64)	0.700

MAP: mean arterial pressure; CI: confidence interval; AUC: area under the curve; EO-PE: early-onset-preeclampsia; LO-PE: late-onset-preeclampsia.
